# One-step ultra-rapid fabrication and thermoelectric properties of Cu_2_Se bulk thermoelectric material

**DOI:** 10.1039/c9ra01008d

**Published:** 2019-04-04

**Authors:** Tiezheng Hu, Yonggao Yan, Si Wang, Xianli Su, Wei Liu, Gangjian Tan, Pierre Poudeu-Poudeu, Xinfeng Tang

**Affiliations:** State Key Laboratory of Advanced Technology for Materials Synthesis and Processing, Wuhan University of Technology Wuhan 430070 China yanyonggao@whut.edu.cn tangxf@whut.edu.cn; Laboratory for Emerging Energy and Electronic Materials (LE^3^M), Department of Materials Science and Engineering, University of Michigan Ann Arbor Michigan 48109 USA

## Abstract

Cu_2_Se is a promising material for high temperature thermoelectric energy conversion due to its unique combination of excellent electronic properties and low thermal conductivity owing to its ionic liquid characteristics at high temperature. In this paper, fully dense single-phase bulk Cu_2_Se material was prepared by the combination of self-propagating high-temperature synthesis (SHS) with *in situ* quick pressing (QP) for the first time. This new approach shortens the duration of the synthesis from days to hours compared to conventional preparation methods. SHS-QP technique is an ultra-fast preparation method, which utilizes the heat released by the SHS reaction and an external applied pressure to achieve the synthesis and densification of materials in one-step. The ultra-fast process of the SHS-QP technique enables the fabrication of single-phase Cu_2_Se bulk materials with relative density of over 98% and with precise control over the stoichiometry owing to the ability to suppress the Se vapor during the reaction. The SHS-QP prepared Cu_2_Se samples exhibit excellent thermoelectric figure of merit, *ZT* ∼ 1.5 at 900 K, which is comparable to those of Cu_2_Se materials prepared by conventional methods. This study opens a new avenue for the ultra-fast and low-cost fabrication of Cu_2_Se thermoelectric materials.

## Abstract 2

Cu_2_Se is a promising material for thermoelectric energy conversion. Fully dense single-phase bulk Cu_2_Se was prepared by the combination of self-propagating high-temperature synthesis with *in situ* quick pressing for the first time.

## Graphical abstract

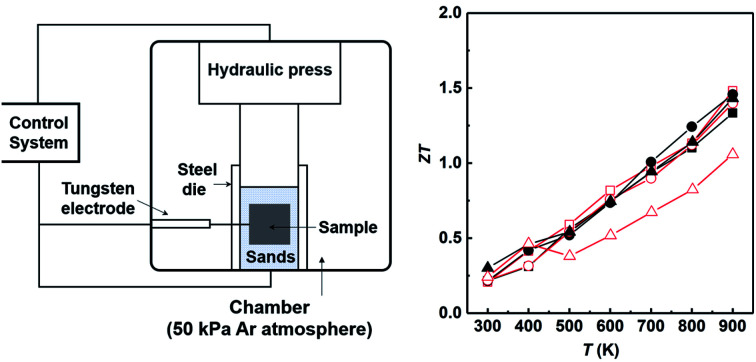

## 1. Introduction

A large increase in the global energy demand along with increasing awareness of environmental pollution has made the development of renewable energy sources of tremendous importance. Along this line, the thermoelectric energy conversion technology has attracted wide attention given its ability to realize a direct and reversible conversion between thermal energy and electric energy using, respectively, the Seebeck effect and Peltier effect of the material. In addition, the thermoelectric technology has the advantages of being contamination-free, noiseless and highly reliable. The thermoelectric properties of a material are characterized by its figure of merit, *ZT* value, which can be defined as *ZT* = *α*^2^*σT*/(*κ*_L_ + *κ*_e_), where *α*, *σ*, *κ*, and *T* are the Seebeck coefficient, the electrical conductivity, the thermal conductivity, and the absolute temperature, respectively. The larger the *ZT* value, the higher the thermoelectric conversion efficiency.^1^

Among the state-of-the-art thermoelectric materials, Cu_2_Se has attracted considerable attention due to the low-cost; Earth abundance and low toxicity of the constituent elements.^2^ The special features of the crystal structure of Cu_2_Se at high temperature are: (1) the rigid face-centered cubic lattice form by Se atoms, which provides a crystalline pathway for holes and hence great electrical properties; and (2) the liquid-like mobility of copper ions arising from the highly disordered copper ions within metal position in the Se sublattice, which enables extremely low lattice thermal conductivity.^3–8^ Therefore, the pristine Cu_2_Se exhibits excellent thermoelectric properties, with *ZT* values as high as 1.5 at 1000 K.^8^

Conventional synthesis methods for Cu_2_Se include melting with long-time annealing,^6,7,9–11^ high-energy ball milling,^12–15^ and solution method.^16^ Some of the disadvantages of conventional synthesis methods that makes it very difficult to get precise control over the chemical composition and hinders large-scale industrial production of Cu_2_Se are long reaction time, high energy consumption, volatility of Se during synthesis and oxidation of Cu. Recently, we have developed a new method to fabricate bulk Cu_2_Se thermoelectric materials, namely the self-propagation high-temperature synthesis (SHS) combined with spark plasma sintering (SPS).^17^ SHS is a technique for rapid preparation of materials by self-exothermic chemical reaction.^18–21^ The preparation of Cu_2_Se thermoelectric material by SHS technique has the advantages of short reaction time and energy saving, simple process, accurate composition control and low cost.^17^ We have synthesized many state-of-the-arts thermoelectric materials through SHS method.^17,22–31^ Although SHS technique has many advantages that traditional preparation methods do not have, the biggest disadvantage is that products obtained are uncompacted.^32,33^ It is still necessary to get fully dense bulk materials by method such as SPS. Therefore, we have improved on the SHS technique by incorporating an *in situ* quick pressing (QP) in order to achieve a fully dense bulk material in just one step. This new technique called “self-propagating high-temperature synthesis combined with quick pressing” (SHS-QP) technique is a simple and economical method to fabricate ceramics. It not only retains the advantages of SHS technology, such as low cost and simple process, but it also overcomes the disadvantage of the uncompact products, which greatly saves the cost.^32,33^ The process of SHS-QP is very simple: a quick and large mechanical force is applied to the sample immediately after the SHS reaction is completed, while the sample is still red-hot and soft. A fully dense bulk material is obtained when the process parameters of SHS-QP are well optimized. Single-phase Cu_2_Se bulk materials with relative density over 98% can be obtained by SHS-QP technique. Due to the ultra-fast SHS process, the volatilization of Se is suppressed and the composition of Cu_2_Se is precisely controlled. The actual composition of Cu_2_Se prepared by SHS-QP in this work is very close to the nominal composition. In addition, the thermoelectric properties of the SHS-QP prepared Cu_2_Se are excellent with the figure of merit, *ZT* ∼ 1.5 achieved at 900 K, which is comparable to those of Cu_2_Se prepared by conventional methods.^8,17^

## 2. Experimental section

### Sample preparation

**Fig. 1:**
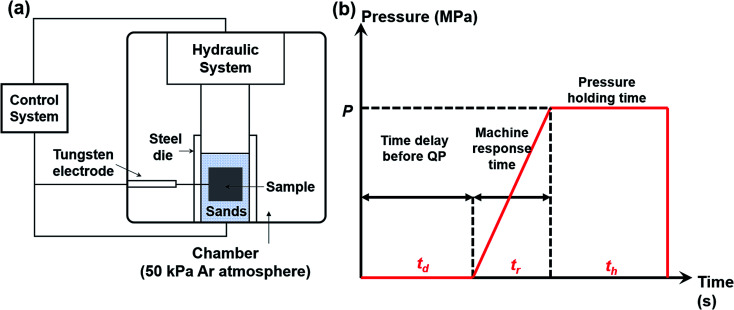
(a) Schematic diagram of the SHS-QP equipment; (b) pressure-time dependence during the SHS-QP process.

Commercially available high-purity powders of Cu (99.99%, <10 μm), Se (99.99%, 200 mesh) were weighed according to a stoichiometric ratio of Cu : Se = 2 : 1 and then mixed uniformly in an agate mortar. The mixtures were cold pressed into pellets with a diameter of 20 mm. The sample pellet was loaded into a customized steel die (*ϕ* = 80 mm) with sands surrounding the pellet. The sands play several roles such as protecting the die, discharging impurity gas, transmitting pressure to the sample and keeping the heat. Then we put the steel die in a customized SHS-QP equipment, as shown in Fig. 1(a). The SHS-QP equipment consists of a hydraulic system, a vacuum system, an ignition system and a control system. The control system regulates the ignition system and the hydraulic system.

Fig. 1(b) shows the classical pressure-time dependence during the SHS-QP process. When the SHS reaction is ignited, a time delay (*t*_d_) before the QP process is needed to ensure that the SHS reaction is completed and the adsorbed impurity gas is fully exhausted. But the time delay cannot be too long because the temperature of the sample drops quickly during the SHS-QP process. The densification will not be achieved when the temperature is too low. It is crucial to get a balance between the exhaustion of impurity gas and the temperature the sample before the QP process. After the time delay, the control system enables the hydraulic system to apply a mechanical force on the sample and the machine needs about 3 s to respond (*t*_r_). When the force is fully applied on the sample, we need to hold the pressure (*P*) for a while (*t*_h_) to ensure that the densification is completed. The pressure should be high enough to activate the plastic flow of the sample.

In a typical experiment, the chamber is filled with 50 kPa of argon gas as protective atmosphere. When the processing parameters, *P*, *t*_d_ and *t*_h_ were well set, the control system bottom is pressed to enable an automatic completion of the whole SHS-QP process. After the complete SHS-QP process, the sample is removed from the die and the surface is polished to remove residual sands and impurities. The sample was then cut into different shapes for various characterizations. Optimal compaction can be obtained by adjusting the densification parameters, *P*, *t*_d_ and *t*_h_.

### Testing and characterization

The actual density of the sample synthesized by SHS-QP was measured by the Archimedes method. The phase purity of the SHS-QP prepared sample (bulk and powder) was characterized by powder X-ray diffraction (PANalytical: Empyrean, Cu Kα) and the microstructure of the sample was observed by FESEM (Hitachi SU8020). The back-scattering electron (BSE) images and the actual composition of the polished surface of the sample were determined from electron probe microanalysis (JEOL, JXA-8230). The combustion temperature of Cu_2_Se during the SHS-QP process was measured by a thermocouple inserted into the center of the sample. The electrical conductivity and the Seebeck coefficient were measured simultaneously by a standard four-probe method (ULVAC-RIKO, ZEM-3) in a helium atmosphere. The thermal conductivity was calculated by the measured thermal diffusivity *D*, specific heat *C*_p_, and density *d* according to the relationship *κ* = *DC*_p_*d*. The thermal diffusivity and the specific heat were respectively measured by laser flash method (NETZSCH, LFA 457) and differential scanning calorimeter apparatus (TA, DSC Q20) under flowing argon atmosphere. All measurements were performed in the temperature range from 300 K to 900 K. The low temperature (10–300 K) Hall coefficient and electrical conductivity measurement were carried out using a Physical Properties Measurement System (Quantum Design, PPMS-9), making use of a five-probe sample configuration while sweeping the magnetic field between −1.0 T and 1.0 T. The carrier concentration (*n*_H_) and the Hall mobility (*μ*_H_) were obtained from the Hall coefficient (*R*_H_) and the electrical conductivity using the relation: *n*_H_ = 1/*e*|*R*_H_| and *μ*_H_ = *σ*|*R*_H_|, respectively, where *e* is the elemental electron charge. The yield strength of the SHS-QP prepared Cu_2_Se was measured by Zwick/Roell (Z005 HT) at 773 K. The variation of the room temperature Seebeck coefficient over the cross section was characterized by a Scanning Seebeck Microprobe (Panco, PSM).

## 3. Result and discussion

**Fig. 2:**
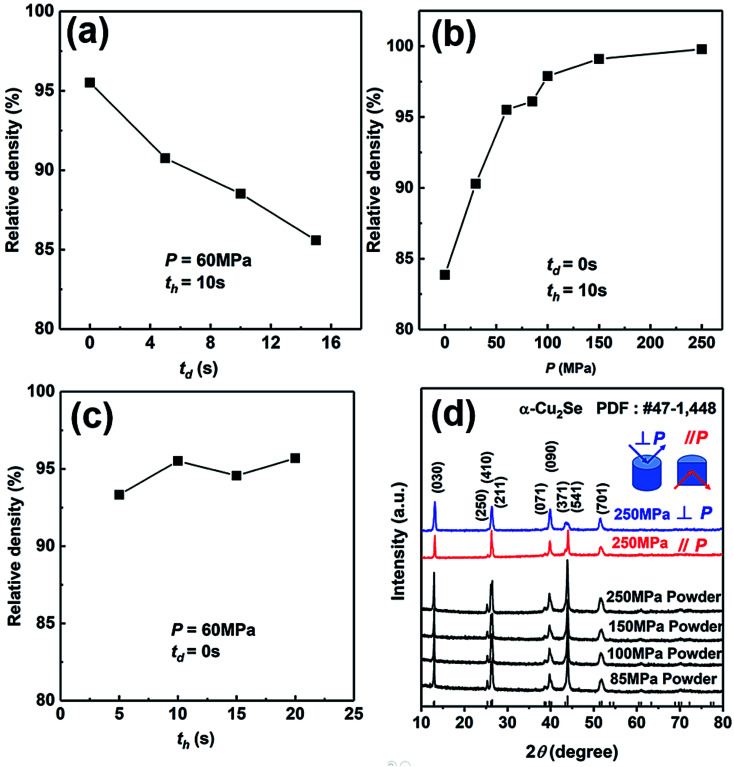
Relative density of the synthesized Cu_2_Se samples as a function of (a) *t*_d_; (b) *P*; and (c) *t*_h_; (d) XRD patterns of SHS-QP prepared Cu_2_Se samples under different pressure and XRD patterns of Cu_2_Se samples along directions perpendicular and parallel to pressing direction when *t*_d_ = 0 s, *t*_h_ = 10 s and *P* = 250 MPa.

We have explored the SHS-QP parameters for the fabrication of high-density bulk Cu_2_Se thermoelectric material. Firstly, we set *P* and *t*_h_ at 60 MPa and 10 s, respectively, to study the effect of *t*_d_ on the density of Cu_2_Se pellet. As we can see in Fig. 2(a), the density of SHS-QP prepared Cu_2_Se sample decreases with increasing *t*_d_. The highest density is achieved at *t*_d_ = 0 s. Since the machine needs 3 s response time before the external force is completely applied on the sample, there is a 3 s window for the completion of the SHS reaction and impurity gas to be exhausted when *t*_d_ = 0 s. Because, the raw materials with high purity are stored in the glovebox, there are just few impurities and adsorbed gas after the SHS-QP process. Therefore, we can obtain a highly dense bulk Cu_2_Se even when *t*_d_ = 0 s.

We then set *t*_d_ and *t*_h_ at 0 s and 10 s, respectively, to study the effect of pressure, *P*, on the relative density of the synthesized Cu_2_Se pellet. As shown in Fig. 2(b), the relative density increases with increasing pressure. When *P* = 0 MPa, the relative density is around 83% and rapidly rise to higher than 97% with *P* > 100 MPa. Further increase in the pressure above 100 MPa leads to very slow increase of the relative density since it is already very close to the theoretical value. Therefore, the optimum pressure should be over 100 MPa.

At last, we set *t*_d_ and *P* at 0 s and 60 MPa, respectively, to study the effect of *t*_h_ on the relative density of Cu_2_Se pellet. As we can see in Fig. 2(c), with the increase of *t*_h_, the change of relative density is not obvious, which means the densification can be completed in a very short time. In conclusion, the optimum densification parameters to achieve relative density of over 97% for bulk Cu_2_Se are: *t*_d_ = 0 s, *P* > 100 MPa and *t*_h_ = 10 s.

We have analyzed the phase of SHS-QP prepared samples under different pressure. Fig. 2(d) shows the XRD patterns of the powders of SHS-QP prepared samples when *t*_d_ = 0 s, *t*_h_ = 10 s and *P* = 85, 100, 150, 250 MPa. The XRD patterns match perfectly with the standard PDF card # 47-1448 with no visible trace of impurities observed, which means that a single phase Cu_2_Se sample can be obtained under any pressure.

We also analyzed XRD patterns taken on samples cut perpendicular and parallel to pressure direction when *t*_d_ = 0 s, *t*_h_ = 10 s and *P* = 250 MPa, and compared them with the theoretical pattern. As we can see in Fig. 2(d), the bulk sample prepared by SHS-QP and cut perpendicular to the pressure direction has preferential orientation on the (0*l*0) plane, while the sample cut parallel to the pressure direction has preferential orientation on the (410) plane. The preferential orientation of the sample perpendicular to pressure direction is stronger than that of the sample parallel to pressure direction. The degree of the grain orientation along (0*l*0) direction can be described by the orientation factor *F*, defined by the following eqn (1)–(3):^23^1
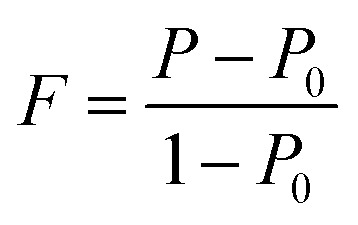
2
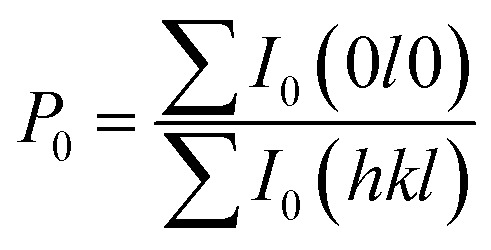
3
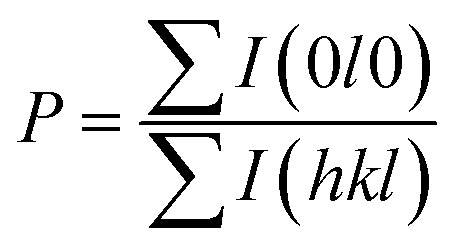
Here, *I* (*hkl*) is the intensity of the (*hkl*) peak of the SHS-QP prepared bulk sample cut perpendicular to the pressure direction, and *I*_0_ (*hkl*) is the intensity of the (*hkl*) peak of the SHS-QP powder having random orientation.^23^ The calculated orientation factor along (0*l*0) plane for sample cut perpendicular to the pressure direction are *F* = 0.26, for Cu_2_Se ingots fabricated when *t*_d_ = 0 s, *t*_h_ = 10 s and *P* = 250 MPa.

**Fig. 3:**
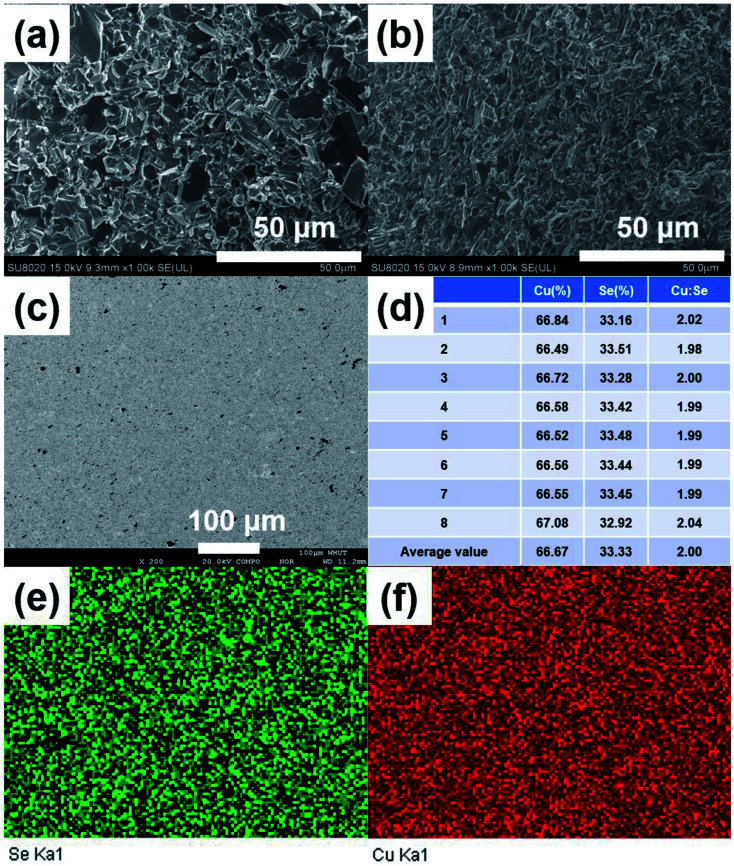
Microstructures of SHS-QP prepared Cu_2_Se when *t*_d_ = 0 s, *P* = 250 MPa and *t*_h_ = 10 s. (a) FESEM images of the fractured surface perpendicular to pressure direction; (b) FESEM images of the fractured surface parallel to pressure direction; (c) BSE image of polished surface; (d) the corresponding EDS results of the same area as in (c); (e) and (f) the corresponding elemental distribution map of the same area as in (c).

Fig. 3(a) and (b) show the FESEM images of the fractured surface of SHS-QP prepared sample when *t*_d_ = 0 s, *t*_h_ = 10 s and *P* = 250 MPa. Fig. 3(a) is the fractured surface perpendicular to pressure direction and Fig. 3(b) is the fractured surface parallel to pressure direction. The grain size varies from 5 μm to 10 μm. We have also calculated the orientation factors of the samples fabricated when *t*_d_ = 0 s, *t*_h_ = 10 s and *P* = 60, 100, 150 MPa. When *P* = 60 MPa, *F* = 0.17; when *P* = 100 MPa, *F* = 0.21; when *P* = 150 MPa, *F* = 0.23. With the increase of pressure, the preferential orientation is enhanced.

Then, we analyzed the actual composition and homogeneity of the SHS-QP prepared Cu_2_Se. Fig. 3(c) shows the BSE image of a polished surface of the sample fabricated when *t*_d_ = 0 s, *P* = 250 MPa and *t*_h_ = 10 s. No contrast difference is observed in the BSE image. Fig. 3(e) and (f) show the elemental distribution map corresponding to Fig. 3(c), both elements Cu and Se are distributed uniformly, indicating that the SHS-QP prepared Cu_2_Se is a homogeneous single phase with no secondary phase appearing on the micrometer scale. We randomly selected 8 points on the polished surface of the sample and analyzed their actual composition by EDS, the results are shown in Fig. 3(d). The actual composition of the sample is very close to the nominal composition 2 : 1, and composition differences between different points are very small. The precise control of the composition comes from the ultrafast temperature rising rate during the SHS process and the high pressure of Ar gas that inhibit the volatilization of Se atom. Therefore, the SHS-QP prepared Cu_2_Se is very homogeneous, and the composition of the sample can be precisely controlled.

The densification mechanism of Cu_2_Se during the SHS-QP process was systematically studied. The SHS-QP process can be considered as a sintering process, which utilizes the heat released by the SHS reaction and external pressure to accomplish the densification.^32–36^ For the densification process of ceramic sintering under pressure, Artz believed that there are three densification mechanism: diffusion, creep and plastic flow.^37^ Among them, diffusion and creep are time and temperature dependent, while plastic flow is time independent.^36,37^ As we discussed above, the densification of SHS-QP can be finished in seconds and the duration of the high temperature is very short. Therefore, the contributions of diffusion and creep to densification are limited. Hence, we believe that plastic flow played a key role in the densification process of Cu_2_Se during SHS-QP.

For ceramics, with lack of slip crystalline feature, plastic flow is realized by plastic yield.^37^ For polycrystalline materials, the yield strength of porous compact is related with both the temperature-dependent theoretical yield strength *σ*_y_ and the relative density *D*. It is determined by the following equations:^37,38^

**Fig. 4:**
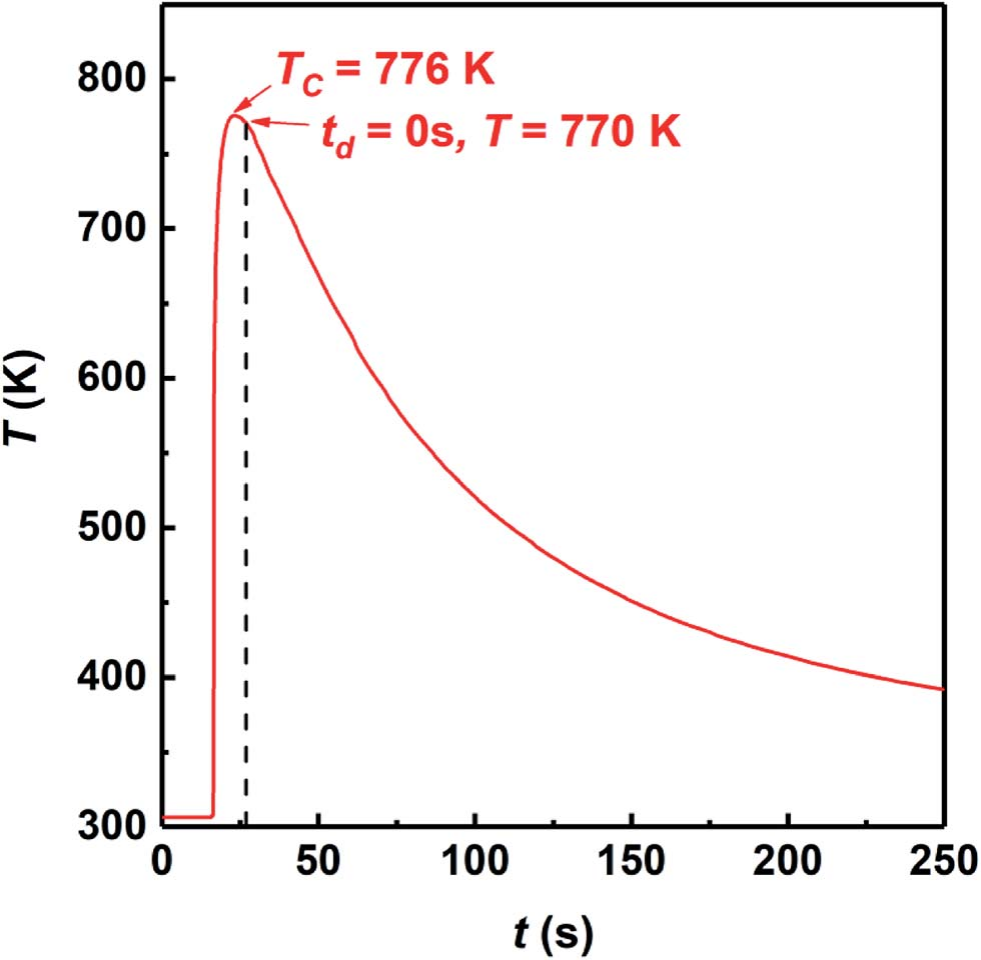
Variation of temperature as a function of time during the SHS-QP process at *P* = 0 MPa.

(1) When *D* < 0.9,4
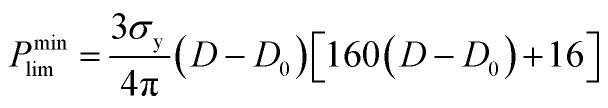


(2) When *D* > 0.9,5
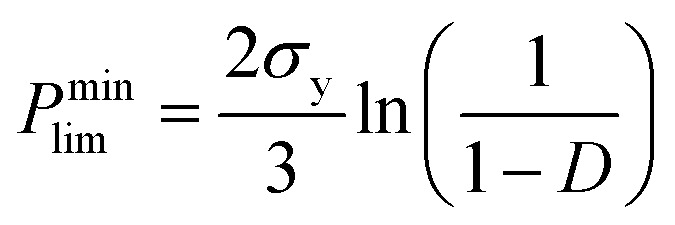
*P*^min^_lim_ is the minimum pressure to activate plastic flow, *D*_0_ and *D* are the initial relative density and transient relative density of the compact, respectively.^37,38^ As shown in Fig. 2(a), the relative density of Cu_2_Se is 84% when *P* = 0 MPa, *D* < 0.9. We measured the combustion temperature of Cu_2_Se in the SHS-QP die when *P* = 0 MPa and the result is shown in Fig. 4. The combustion temperature *T*_c_ can reach 776 K. Considering the 3 s machine response time, the temperature of sample drops to 770 K when the pressure is completely applied to the sample. We measured the yield strength of the SHS-QP prepared Cu_2_Se at 770 K, which is about 45 MPa. Therefore, we can calculate the minimum pressure to activate plastic flow at 770 K from eqn (4), *P*^min^_lim_ = 95 MPa. This means that during the SHS-QP process, when *t*_d_ = 0 s, the external pressure should be higher than 95 MPa to activate the plastic of Cu_2_Se. As we can see in Fig. 2(b), the relative density of Cu_2_Se can reach over 97% when *P* > 100 MPa, which is consist with the calculation result.

**Fig. 5:**
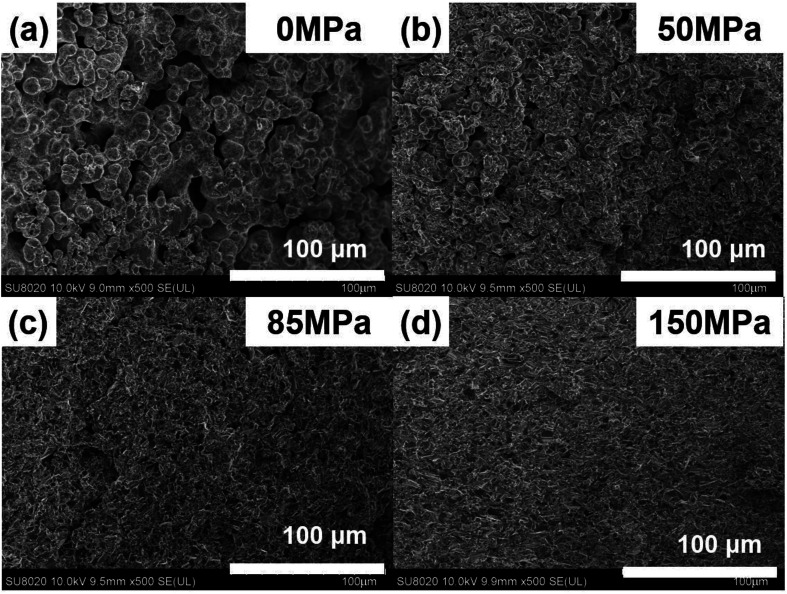
FESEM images of the fractured surface of the SHS-QP prepared Cu_2_Se when *t*_d_ = 0 s, *t*_h_ = 10 s and under different pressure: (a) *P* = 0 MPa; (b) *P* = 50 MPa; (c) *P* = 85 MPa; (d) *P* = 150 MPa.

Fig. 5 shows the FESEM images of Cu_2_Se under different pressure when *t*_d_ = 0 s and *t*_h_ = 10 s. Fig. 5(a) shows the microstructure of the SHS-QP prepared Cu_2_Se when *P* = 0 MPa. At this stage, the SHS reaction is already finished while the force has not yet been applied to the sample. It can be observed that the sample is at the early stage of sintering, the shape of the particles is spherical, most particles begin to form a neck connection, and the pores are all interconnected. Since there is no external pressure at this stage, the densification mechanism at this stage is mainly dominated by diffusion.

Fig. 5(b) shows the microstructure of the SHS-QP prepared Cu_2_Se when *P* = 50 MPa. The particles are still spherical in shape, the sample is in the middle stage of sintering. The particles have been rearranged, mass transfer started, and grain boundaries have increased, the pores are greatly reduced. The external pressure at this stage cannot activate the plastic flow; therefore, the densification mechanisms at this stage are mainly dominated by diffusion and particle rearrangement.

Fig. 5(c) shows the microstructure of the SHS-QP prepared Cu_2_Se when *P* = 85 MPa. It can be observed that the sample is in the last stage of sintering. The shape of the particles has changed from sphere to irregular polygon with a layered structure. The change of particle morphology is due to the plastic yielding under the external force. At this stage, the plastic flow has been activated, huge pores have been basically removed, the density of the sample has been largely increased, but there are still a few small pores left. The dominant densification mechanism at this stage is plastic flow since the pressure of 85 MPa is very close to the calculated minimum pressure to activate plastic flow (95 MPa).

Fig. 5(d) shows the microstructure of the SHS-QP prepared Cu_2_Se when *P* = 150 MPa. The particles are mainly irregular polygons in shape with layered structures, the pores have been removed and the sample is highly dense.

In brief, the densification process of Cu_2_Se during the SHS-QP process consists of rapid temperature rise to 773 K after the SHS reaction, the initiation of diffusion between particles and formation of necks interconnecting various particle. When the pressure is applied on the sample, the particles are rearranged, pores begin to reduce; and when the pressure is high enough to make Cu_2_Se particles plastically yield, the plastic flow is activated, and the shape of the particles change from spherical to irregular polygons with layered structure. At this stage the pores have been removed and the relative density of the sample is very high indicating completion of the densification process.

**Fig. 6:**
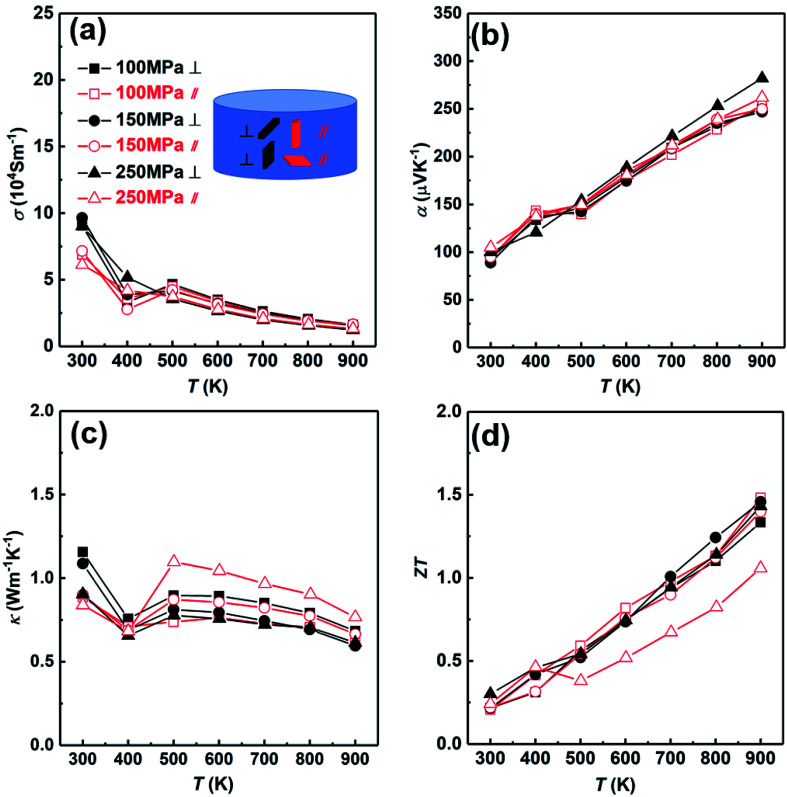
Thermoelectric properties of SHS-QP synthesized Cu_2_Se samples (when *t*_d_ = 0 s, *t*_h_ = 10 s and *P* = 100, 150, 250 MPa) cut in different directions (perpendicular (⊥) and parallel (∥)): (a) electrical conductivity; (b) Seebeck coefficient; (c) thermal conductivity; (d) *ZT* value.

Then we analyzed the thermoelectric properties of the SHS-QP prepared Cu_2_Se. Due to the existence of preferred orientation in the sample prepared by SHS-QP, and the preferred orientation is pressure dependent, we studied the effects of orientation and pressure on thermoelectric properties. Fig. 6 shows the thermoelectric properties of different directions when *t*_d_ = 0 s, *t*_h_ = 10 s and *P* = 100 MPa, 150 MPa, 250 MPa. The thermoelectric properties of samples cut perpendicular and parallel to pressure direction are represented by black lines and red lines, respectively. Fig. 6(a) shows the temperature dependent electrical conductivity. When 300 K < *T* < 400 K, the electrical conductivities in both directions with different pressure all decrease with the increasing temperature. The electrical conductivities of the samples cut perpendicular to pressure direction are slightly higher than that of the samples cut parallel to pressure direction under the same pressure. When 400 K < *T* < 500 K, the electrical conductivities in both directions with different pressure all increase with the increasing temperature due to the α to β phase transition of Cu_2_Se near 440 K. When *T* > 500 K, the electrical conductivities in both directions with different pressure all decrease with the rising temperature again, showing a heavy doped semiconducting characteristic. This result is consistent with isotropic physical properties expected due to the cubic crystal structure of Cu_2_Se within this temperature range. The electrical conductivities vary little with the increasing pressure in both directions. Fig. 6(b) shows the temperature dependent Seebeck coefficient. The Seebeck coefficients in both directions under any pressure are positive in the whole temperature range, indicating a p-type conducting behavior. They increase with the increasing temperature and fluctuate around 400 K due to the α to β phase transition of Cu_2_Se. The room temperature Seebeck coefficient values of all samples are very close, which are around 100 μV K^−1^. The Seebeck coefficient of the sample cut perpendicular to the pressure direction and under 250 MPa pressure is slightly higher than other samples at high temperature (*T* > 600 K). Besides that, the Seebeck coefficients of the other samples in both directions with different pressure are very close in the whole temperature range. Fig. 6(c) shows the temperature dependent thermal conductivity. The thermal conductivities of all samples first decrease with increasing temperature from 300 K to 400 K, then increase with increasing temperature until 500 K due to the α to β phase transition of Cu_2_Se, at last decrease with increasing temperature when *T* > 500 K. For the samples cut parallel to the pressure direction, the thermal conductivities of samples are almost the same under different pressure when 300 K < *T* < 400 K, and increase with the increasing pressure when *T* > 400 K. For the samples cut perpendicular to the pressure direction, the thermal conductivities of samples under different pressure decrease with the increasing pressure at the whole temperature range. When *P* = 100 MPa, the thermal conductivity of the sample cut perpendicular to the pressure direction is higher than that of the sample cut parallel to the pressure direction in whole temperature range. When *P* = 150 MPa and 250 MPa, the thermal conductivities of the samples cut perpendicular to the pressure direction are higher than that of the samples cut parallel to the pressure direction when *T* < 400 K. However, when *T* > 400 K, the thermal conductivities of the samples cut perpendicular to the pressure direction become lower than that of the samples cut parallel to the pressure direction. Fig. 6(d) shows the temperature dependent *ZT* value. When *P* = 100 MPa, due to the lower thermal conductivity of the sample cut parallel to the pressure direction in the whole temperature range, the *ZT* value of the sample cut parallel to pressure direction is slightly higher than that of the sample cut perpendicular to pressure direction and reaches 1.5 at 900 K. When *P* = 150 MPa and 250 MPa, due to the higher electrical conductivities at low temperature (*T* < 400 K) and the lower thermal conductivities of the samples cut perpendicular to the pressure direction at high temperature (*T* > 400 K), the *ZT* values of the samples cut perpendicular to the pressure direction are higher than that of the samples cut parallel to pressure direction. When *P* = 150 MPa and 250 MPa, *ZT* values of the samples cut perpendicular to the pressure direction as high as 1.46 and 1.45 were obtained at 900 K, respectively. The *ZT* values of the samples prepared by SHS-QP are higher than the *ZT* value of 1.2 reported at 900 K for Cu_2_Se sample prepared by the traditional melt-annealing combined with SPS sintering method,^8^ and is comparable to the *ZT* value of 1.45 at 900 K measured for Cu_2_Se sample prepared by SHS-SPS.^17^

**Fig. 7:**
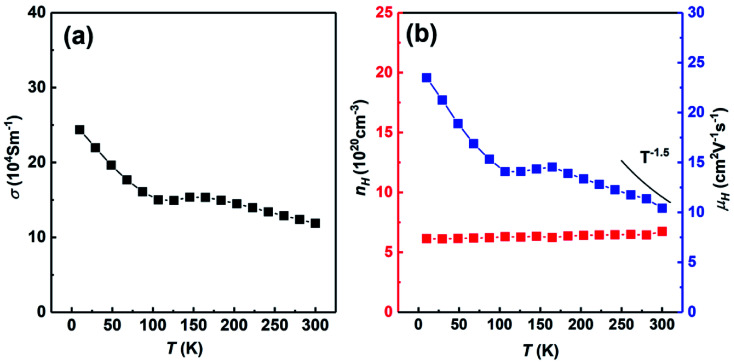
Temperature dependent electrical conductivity, carrier concentration and carrier mobility of SHS-QP prepared sample (*t*_d_ = 0 s, *P* = 250 MPa and *t*_h_ = 10 s) cut perpendicular to the pressure direction: (a) electrical conductivity; (b) carrier concentration and carrier mobility.

Fig. 7 shows the temperature dependent electrical conductivity, carrier concentration and carrier mobility of SHS-QP fabricated Cu_2_Se sample (when *t*_d_ = 0 s, *P* = 250 MPa and *t*_h_ = 10 s) cut perpendicular to the pressure direction. Fig. 7(a) shows the temperature dependent electrical conductivity at low temperature. The electrical conductivity decreases with the increasing temperature at whole temperature range and reaches around 11 × 10^4^ S m^−1^ at 300 K, which is consisted with the room temperature electrical conductivity measured by ZEM. Fig. 7(b) shows the temperature dependent carrier concentration and carrier mobility. The carrier concentration is roughly temperature independent in the whole temperature range. The room temperature carrier concentration reaches 6.7 × 10^20^ cm^−3^. The carrier mobility roughly decreases with increasing temperature. The temperature dependence of the carrier mobility follows the power law *T*^−3/2^ around room temperature, implying a scattering mechanism dominated by acoustic phonon scattering. The room temperature carrier mobility reaches 10.4 cm^2^ V^−1^ s^−1^.

We have compared the room temperature electrical properties of Cu_2_Se prepared by different methods, the results are shown in Table 1. The carrier concentration of SHS-QP prepared Cu_2_Se (6.7 × 10^20^ cm^−3^) is almost twice as high as the facile solution combined with SPS (FS-SPS) prepared Cu_2_Se (3 × 10^20^ cm^−3^),^16^ which brings the electrical conductivity of SHS-QP prepared Cu_2_Se (11 × 10^4^ Sm^−1^) twice as high as electrical conductivity of the FS-SPS prepared Cu_2_Se (3 × 10^4^ Sm^−1^). On the other hand, a higher carrier concentration leads to a lower the Seebeck coefficient, the room temperature Seebeck coefficient of SHS-QP prepared Cu_2_Se (100 μV K^−1^) is 20% lower compared with the FS-SPS prepared Cu_2_Se (120 μV K^−1^). While compared with the high-pressure technology at room temperature (HPRT) prepared Cu_2_Se,^39^ the carrier concentration of SHS-QP prepared Cu_2_Se is slightly lower and the carrier mobility is almost the same, which brings the slightly lower electrical conductivity and relatively higher Seebeck coefficient.

**Table 1:** Room temperature electrical properties of Cu_2_Se prepared by different method

Method	*σ* (10^4^ S m^−1^)	*α* (μV K^−1^)	Carrier concentration (10^20^ cm^−3^)	Carrier mobility (cm^2^ V^−1^ s^−1^)
SHS-QP	11	100	6.7	10.4
FS-SPS^16^	6.68	120	3	—
HPRT^39^	13.1	68	7.7	10.7

**Fig. 8:**
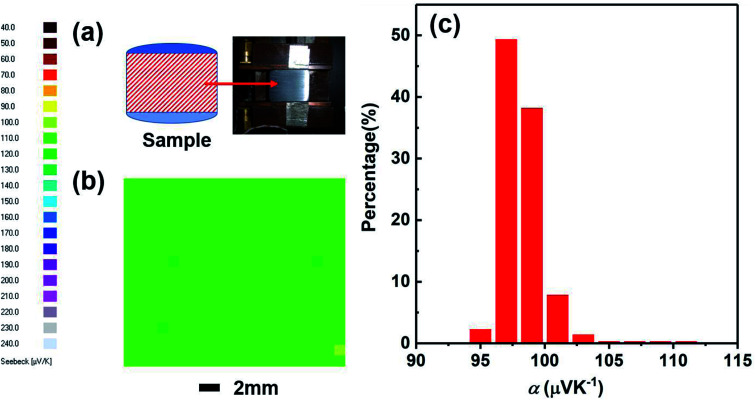
(a) Image of the cross section of SHS-QP prepared Cu_2_Se sample where the Seebeck coefficient data were measured; (b) distribution of the room temperature Seebeck coefficient values on the cross section of the sample when *t*_d_ = 0 s, *P* = 250 MPa and *t*_h_ = 10 s; (c) statistical distribution of Seebeck coefficients on the cross section.

We have studied the homogeneity of the SHS-QP prepared Cu_2_Se bulk material. We selected the SHS-QP prepared Cu_2_Se sample obtained under the conditions, *t*_d_ = 0 s, *P* = 250 MPa and *t*_h_ = 10 s, and cut into two parts in the direction parallel to the pressing direction (Fig. 8(a)). Fig. 8(b) shows the distribution of the room temperature Seebeck coefficient values measured over the cross section of SHS-QP prepared Cu_2_Se (when *t*_d_ = 0 s, *P* = 250 MPa and *t*_h_ = 10 s). As we can see, the distribution of Seebeck coefficients is very uniform, and the Seebeck coefficient value is around 100 μV K^−1^, which is consistent with the Seebeck coefficient value measured by the commercial ZEM apparatus. Fig. 8(c) shows the statistical distribution of the Seebeck coefficient values on the cross section. Most Seebeck coefficient values are distributed in the range of 97–103 μV K^−1^, indicating that the sample is very homogenous.

**Fig. 9:**
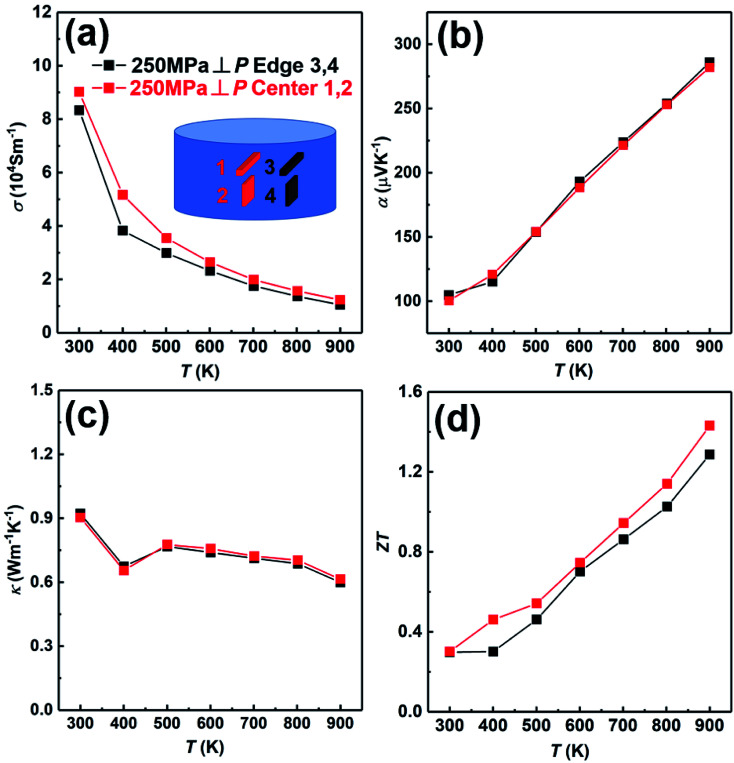
Thermoelectric properties of the center part and edge part of the sample prepared under the conditions; *t*_d_ = 0 s, *P* = 250 MPa and *t*_h_ = 10 s: (a) electrical conductivity; (b) Seebeck coefficient; (c) thermal conductivity; (d) *ZT* value.

In order to further prove the uniformity of the sample, we have measured the thermoelectric properties of the center part and edge part of the sample fabricated under the condition; *t*_d_ = 0 s, *P* = 250 MPa and *t*_h_ = 10 s, as shown in Fig. 9. The electrical conductivity of the center part is slightly higher than that of the edge part in whole temperature (Fig. 9(a)). The Seebeck coefficients of both center part and edge part are almost the same (Fig. 9(b)). The thermal conductivity of both center part and edge part are also almost the same (Fig. 9(c)). Due to the slightly higher electrical conductivity, the *ZT* value of the center part is slightly higher than that of the edge part (Fig. 9(d)). In conclusion, the thermoelectric properties of the center part and edge part of the sample are almost the same, indicating the excellent homogeneity of the SHS-QP prepared bulk Cu_2_Se sample.

**Fig. 10:**
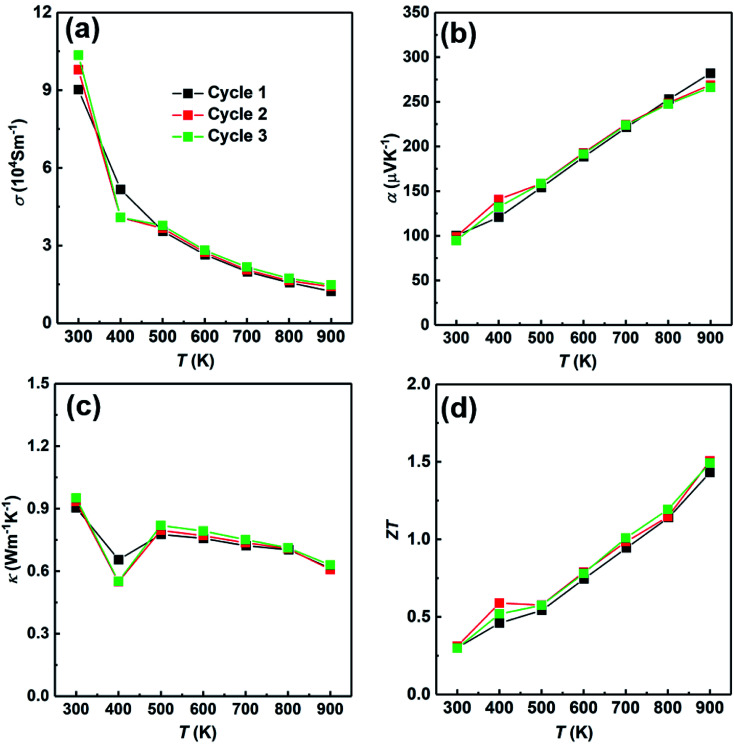
Thermoelectric properties of the SHS-QP prepared sample (*t*_d_ = 0 s, *P* = 250 MPa and *t*_h_ = 10 s, along the perpendicular to pressure direction) under 3 heating cycles: (a) electrical conductivity; (b) Seebeck coefficient; (c) thermal conductivity; (d) *ZT* value.

At last, we have studied the stability of the SHS-QP prepared sample. We have measured the sample prepared under the conditions; *t*_d_ = 0 s, *P* = 250 MPa and *t*_h_ = 10 s and cut perpendicular to pressure direction for 3 times to study the stability of the SHS-QP prepared sample, the results are shown in Fig. 10. The electrical properties and thermal property changed very little after going through high temperatures, which indicates the stability of the SHS-QP prepared Cu_2_Se.

## 4. Conclusions

In this study, fully dense single-phase Cu_2_Se bulk material was prepared by self-propagating high-temperature synthesis (SHS) followed by *in situ* quick pressing (QP) for the first time. When *t*_d_ = 0 s, *P* > 100 MPa and *t*_h_ = 10 s, bulk Cu_2_Se material with relative density higher than 97% is obtained. The densification process of Cu_2_Se during SHS-QP is divided in to 3 stages: diffusion, particle rearrangement and plastic flow. The plastic flow stage plays a key role in the densification process.

Due to the ultra-fast SHS process, the composition of Cu_2_Se can be precisely controlled. It was found that the actual composition of the SHS-QP prepared Cu_2_Se is very close to the nominal composition. The homogeneity and stability of the SHS-QP prepared Cu_2_Se are very good.

Due to the high temperature gradient and high pressure during SHS-QP process, the bulk sample prepared by SHS-QP has preferential orientation. Along perpendicular to pressure direction the sample has a preferential orientation on the (0*l*0) plane, while along parallel to pressure direction the sample has a preferential orientation on the (410) plane. The preferential orientation on the (0*l*0) plane brings the reduction on thermal conductivity when *T* > 400 K while the preferential orientation on the (410) plane brings the increment on thermal conductivity when *T* > 400 K.

The *ZT* value of Cu_2_Se sample fabricated under the condition; *t*_d_ = 0s, *P* = 100 MPa and *t*_h_ = 10 s, and cut parallel to the pressure direction reaches 1.5 at 900 K, which is higher than the *ZT* value of 1.2 at 900 K reported for Cu_2_Se sample prepared by the traditional melt-annealing combined with SPS sintering method,^8^ and is comparable to the *ZT* value 1.45 at 900 K reported for Cu_2_Se sample prepared by SHS-SPS.^17^

Compared with traditional methods, the preparation of Cu_2_Se thermoelectric material by SHS-QP technique has the advantage of reducing the fabrication time from days to hours. In addition, this new technique is simple, requires considerably lower energy and enables precise control over the composition. This study opens a new avenue for ultra-fast and low-cost fabrication of Cu_2_Se thermoelectric materials.

## Conflicts of interest

There are no conflicts of interest to declare.

## Supplementary Material
